# A System for Creating Stable Cell Lines that Express a Gene of Interest from a Bidirectional and Regulatable Herpes Simplex Virus Type 1 Promoter

**DOI:** 10.1371/journal.pone.0122253

**Published:** 2015-03-30

**Authors:** Christopher B. Chambers, William P. Halford, Joshua Geltz, Olga Villamizar, Jeffrey Gross, Alison Embalabala, Edward Gershburg, Andrew Wilber

**Affiliations:** Department of Medical Microbiology, Immunology and Cell Biology, Southern Illinois University School of Medicine, Springfield, Illinois, United States of America; Friedrich-Loeffler-Institute, GERMANY

## Abstract

Expression systems used to study the biological function of a gene of interest can have limited utility due to three major factors: i) weak or heterogeneous gene expression; ii) poorly controlled gene expression; and iii) low efficiencies of stable integration and persistent expression. We envisioned that the ideal system should be tightly controlled and coupled with the ability to efficiently create and identify stable cell lines. Herein, we describe a system based upon a bidirectional Herpes simplex virus type 1 promoter that is naturally responsive to the VP16 transactivator and modified to permit tetracycline-regulated transcription on one side while maintaining constitutive activity on the other side. Incorporation of this element into the *Sleeping Beauty* transposon resulted in a novel bidirectional system with the capacity for high-efficiency stable integration. Using this system, we created stable cell lines in which expression of a gene of interest was tightly and uniformly controlled across a broad range of levels via a novel combination of doxycycline-sensitive de-repression and VP16-mediated sequence-specific induction. The unique characteristics of this system address major limitations of current methods and provide an excellent strategy to investigate the effects of gene dosing in mammalian models.

## Introduction

The ability to manipulate gene expression either through overexpression or knockdown is necessary to study the biological function of a gene of interest. However, current expression systems can have limited utility due to three major factors: i) weak or heterogeneous gene expression; ii) poorly controlled gene expression; and iii) low efficiencies of stable integration and persistent expression. These are critical limitations as the amount of a particular gene product can influence nearly every cellular process. Fortunately, the effects of gene dosage can be studied using strategies developed to keep gene expression "off" or "on" when a chemical or factor is introduced into the culture media or animal. The most well-known gene regulation systems are based on the principle of tetracycline (Tet) dependent transcription [1], and consist of two components: (i) an activator or repressor protein, which can be modulated by the addition of Tet or doxycycline (Dox), and (ii) a promoter which is dependent on the binding of the activator or repressor.

Tet-regulated systems have the capacity to permit defined and reversible changes in gene activity. However, optimal performance requires that the activator or repressor be present at a certain intracellular concentration, and that the promoter and gene of interest be inserted in a region of the genome that does not interfere with promoter function. The latter point is highlighted by studies demonstrating that a Tet-regulated version of the human cytomegalovirus (hCMV) immediate-early promoter was susceptible to activation from genomic enhancer sequences located near the site of integration resulting in “leaky” or poorly controlled transcription [1]. Similarly, the ability of the activator to enhance transcription was also impacted by the site of genomic integration [1]. Follow-up studies did reveal the existence of genomic sites where the Tet-responsive hCMV promoter exhibited essentially no activity in the uninduced state but high-level transcription when induced. However, these sites made up only about 5–15% of the cumulative integration events for stably transfected cells [2]. These collective reports indicated that there is clear variation in basal promoter activity for inducible expression systems.

In these early studies, gene delivery was achieved by cloning the inducible expression cassettes into plasmids which were transfected into cells. Coexpression of a selectable gene product, in this case a drug resistance gene, from a second constitutive promoter permitted the outgrowth of stably transfected cell populations. While still frequently used today, this method of generating cell lines is highly inefficient because it relies upon random, non-homologous integration into chromosomes. Alternatively, a few non-viral systems have the capacity for integration and long-term gene expression via a cut-and-paste mechanism; such is possible with the *Sleeping Beauty* transposon [3]. *Sleeping Beauty* (SB) mediates chromosomal integration and stable gene expression when an SB transposon containing a genetic cargo is co-delivered along with the catalytic transposase that is supplied on the same (*cis*) or separate (*trans*) plasmid from the transposon. When expressed, the transposase binds to direct repeat (DR) sequences within the transposon ends, removes the element from the donor plasmid, and precisely inserts the sequences into the genome at a TA-dinucleotide target site [4]. Using the most active versions of transposase, stable gene transfer efficiencies compare favorably with integrating viral vectors [5].

Having experienced the aforementioned limitations of inducible expression systems in our studies, we were interested in developing a system that utilized a single promoter capable of providing inducible control of a gene of interest and constitutive expression of a marker gene. With this goal in mind, we focused our attention on bidirectional promoters which have the ability to direct coordinate expression of multiple genes [6–8]. Researchers have constructed synthetic bidirectional promoters that incorporate Tet-responsive elements to direct expression of two genes [8,9]. However, these synthetic promoters could not limit control to a single side of the promoter and required extensive cloning efforts for construction.

Our approach was to combine the function of a naturally occurring bidirectional promoter with Tet/Dox regulation and transposon gene delivery to create a novel system that allows for easy visual screening of successfully transfected clones and also demonstrates expression of a gene of interest ranging from none (or background levels) to high. For this, we cloned a bidirectional immediate early (IE) promoter from the Herpes simplex virus type 1 (HSV-1) genome which included six VP16 response elements that can be exploited to induce gene expression over basal levels when this activator protein is present. The HSV-1 IE promoter was modified by introducing two tetracycline response elements (2xOp) to one side (5’ end) to provide an additional level of control via Dox-regulated gene expression. We introduced two reporter genes, green fluorescent protein (GFP) and a truncated form of the low affinity human nerve growth factor receptor (NGFR) [10], on the 5’ and 3’ ends of the IE promoter, respectively, and inserted the cassette into an SB transposon. We compared gene expression between our system and a commercially available inducible system (T-REx; Life Technologies) in a cell line that stably expressed the Tet-repressor protein. These experiments revealed that the commercial system had limited capacity for generating tightly regulated cell lines (<15% efficiency). Alternatively, the majority of cell lines generated using the bidirectional IE system had low to undetectable GFP expression in the basal state. Addition of Dox resulted in a homogenous increase in GFP expressing averaging nearly 10-fold above background and this level was significantly higher after Dox plus VP16 treatment, ranging up to nearly 100-fold above baseline levels. Further enhancements included the development of a second transposon that conferred high-level expression of a bicistronic transcript encoding for the Tet-repressor protein and puromycin resistance gene product. With this refinement, we demonstrate the ability to generate cells lines with regulated and broad-range expression of an influenza virus hemagglutinin (HA) protein. The unique characteristics of this system address major limitations of current methods and provide an excellent strategy to investigate the effects of gene dosing in mammalian models.

## Materials and Methods

### Vector construction

#### TRP-GFP Plasmid

A GFP coding sequence was PCR amplified from pEGFP-C1 (Clontech) using primers: GFP for: 5’- GAT CCA TGG TGA GCA AGG GCG-3’and GFP rev: 5’- CAT CTC GAG TTA CTT GTA CAG CTC GTC C-3’, which included recognition sequences for *Nco*I and *Xho*I (underlined) at the 5’- and 3’-ends of GFP. PCR reactions were performed using Pfu Taq polymerase and conditions: 98°C-2 min followed by 35 cycles at 98°C-30 sec, 58°C-30 sec, 72°C-1 min with a final extension at 72°C for 10 min before terminating at 4°C. The resulting product was gel purified, digested with *Nco*I and *Xho*I and inserted into pFastBac (Invitrogen) digested with the same enzymes to create pFastBac-GFP. A *Bam*HI to *Xho*I fragment encoding GFP was recovered and inserted into the pcDNA5/TO (Life Technologies) that was similarly digested. Ligation created TRP-GFP encoding for GFP expression under control of a tetracycline responsive version of the cytomegalovirus (CMV) promoter and positioned upstream of a simian virus (SV) 40 promoter regulated blasticidin resistance gene.

*Sleeping Beauty* transposon vectors were constructed using T2 inverted terminal repeat sequences as described [11] and co-delivered with transposase (SB11) encoding plasmids in which expression was regulated by the human phosphoglycerate kinase (PGK) promoter termed PGK-SB11 [12].

#### i. TRP-GFP

The tetracycline-regulated GFP expression cassette was excised from TRP-GFP by digestion with *Mfe*I and overhangs filled-in with Klenow DNA polymerase followed by digestion with *Pvu*II (blunt). The resulting 1869-bp fragment encoding for the tetracycline responsive CMV promoter, GFP and bovine growth hormone (BGH) polyadenylation signal was inserted into the transposon vector pKT2/SE digested with *Pme*I (blunt) and dephosphorylated with calf alkaline phosphatase (CIP). Ligation created a transposon encoding for tetracycline regulated expression of GFP.

#### ii. G-MCS-N

The GFP coding sequence was PCR amplified from TRP-GFP using primers GFP linker for: 5’ACG CGT TCT CCG GAC TAG ATC TAA CTG CAG CAC TAG TCG GAT CCA CCG GTC GCC ACC ATG GTG AGC AAG GGC GAG GAG C-3’and GFP linker rev: 5’-GCA TGG ACG AGC TGT ACA AGT AAA GCG GCC GTC TAG ACC GCG GCC GCC TGA CGT CGC GGG TAA CCA CGG TCG ACA T-3’. PCR reactions were performed with Phusion Hi-Fidelity Taq polymerase (Fermentas) and conditions: 98°C-2 min followed by 35 cycles at 98°C-30 sec, 58°C-30 sec, 72°C-1 min with a final extension at 72°C for 10 min before terminating at 4°C. The resulting 830-bp product was gel purified, A-tailed and introduced into pCR2.1 TOPO/TA to create pCR2.1/GFP linker and sequence verified (GenScript). A *Sac*I to *Sal*I fragment encoding the linker-modified GFP sequences was recovered and ligated into a transposon-encoding for NGFR followed by an SV40 poly-adenylation signal that was digested the same enzymes. This created G-MCS-N where GFP and NGFR were separated by unique restriction sites for *Mlu*I, *Bsp*EI, *Bgl*II, *Pst*I, *Spe*I, and *Bam*HI (underlined above).

#### iii. G-C-N

A pGEM-2 plasmid encoding sequences for the human cytomegalovirus (CMV) immediate early promoter-enhancer [13] was digested with *Pst*I to release a 2100-bp fragment that was introduced into G-MCS-N digested with *Pst*I and dephosphorylated with CIP. Ligation created transposon-based expression vectors with the CMV promoter in both sense and antisense orientations and activity monitored based on expression of GFP and NGFR.

#### iv. G-IE-N

The plasmid p0+GFP24 [14] was digested with *Bgl*II and *Bsp*EI to recover 1724-bp of HSV-1 genomic DNA encoding for the 736-bp bidirectional promoter including the six VP16 response elements and 761-bp of sequences from the noncoding intron 1 of ICP0. This fragment was cloned into *Bgl*II-*Bsp*EI digested G-MCS-N to create G-IE-N.

#### v. Tetracycline inducible versions of G-IE-N

The HSV IE bidirectional promoter in G-IE-N was modified to include two tandem copies of Tet operator sequences (2xOp or TR) at the 5’ (ICP0) end of the promoter near the TATA box (G-IE-N(TR^TATA^)) or within the non-coding intron located immediately upstream of GFP (G-IE-N(TR^Intron^)). To construct these vectors, two 227-bp oligonucleotides were created that when annealed encoded for a 5’-*Bsu*36I site followed by 160-bp of sequences (homologous to either the promoter or non-coding intron), two binding sites (underlined) for the Tet repressor protein GG GAT AGT CAC TAT CTC TAG AGG GAT AGT CAC TAT C and an additional 28-bp before terminated with a *Nhe*I site. Ligation of these sequences into *Bsu*36I-*Bgl*II digested G-IE-N created two versions that of the promoter that were tested for response to tetracycline.

#### vi. Tetracycline inducible HA-IE-N

A pCEP4 plasmid encoding sequences for the influenza virus A hemagglutinin (HA) protein (strain Puerto Rico/8/1934), a kind gift from Dr. Tom Griffith, University of Minnesota, was digested sequentially with *Hin*dIII and *Not*I and overhangs filled-in with Klenow DNA polymerase to create blunt ends. The resulting 1741-bp fragment was inserted into G-IE-N(TR^TATA^) digested with *Xba*I and *Bgl*II and treated with Klenow DNA polymerase before being dephosphorylated with CIP. Ligation created a transposon encoding for tetracycline regulated expression of HA.

#### vii. Tetracycline Repressor (TetR) Transposon

The TetR coding sequences were PCR amplified from pcDNA6/TR (Life Technologies) using primers TetR for: 5’- CAA TTG GTA ATA CGA CTC ACT ATA GG -3’ and TetR rev: 5’- CAA TTG GTA ACC ATT ATA AGC TGC -3’designed to introduce recognition sequences for *Mfe*I (underlined) at the 5’- and 3’-termini. PCR reactions were performed with Phusion Hi-Fidelity Taq polymerase (Fermentas) and conditions: 95°C-1 min followed by 35 cycles at 95°C-30 sec, 61°C-30 sec, 72°C-1 min with a final extension at 72°C for 10 min before terminating at 4°C. The resulting 734-bp product was gel purified, A-tailed and introduced into pCR2.1 TOPO/TA to create pCR2.1/TetR and sequence verified (GenScript). An *MfeI* to *MfeI* fragment encoding the TetR coding sequence was recovered and inserted into a transposon-encoding pKT2/Cags-Luc-ires-Puro digested with *Eco*RI to remove coding sequences for firefly luciferase before being dephosphorylated with CIP. Ligation with TetR created pKT2/Cags-TetR-ires-Puro.

#### DNA Preparation

All plasmids used in transfections were prepared using Endotoxin-free Maxi Prep (Qiagen).

### Cell culture, transfection and selection of drug-resistant colonies

Human embryonic kidney (HEK) 293T and HeLa cervical carcinoma cells were purchased from American Type Culture Collection (ATCC). Both lines were **cultured** in Dulbecco’s modified Eagle medium (DMEM) supplemented with 10% fetal bovine serum (FBS), and 1% penicillin-streptomycin at 37°C in a humidified atmosphere containing 5% CO_2_. For transfection, 3–4 x 10^5^ cells were seeded into 6-well tissue culture dishes and allowed to adhere overnight. The next day medium was removed and 1 mL of OptiMEM (Invitrogen) containing Lipofectamine 2000 (Invitrogen) complexed DNA added drop-wise to the cells. After 3–4 hours of incubation, the transfection medium was replaced with fresh growth medium. Two days later, viable cells (trypan blue negative) were serially diluted 1:3 to achieve 100,000 to 300 total cells in 100-mm dishes containing growth medium supplemented with either blasticidin (10 μg/mL), hygromycin (100 μg/mL), or puromycin (0.5 μg/mL). After 12–14 days of selection, well-isolated, drug-resistant colonies were removed from the plates using borosilicate glass cloning cylinders (Bellco, Vineland, NJ) and selectively expanded to generate single cell-derived cell lines.

### Generation of stable cell lines

#### i. Tetracycline Repressor (TetR)

Cells with stable expression of TetR protein were created by transfecting HEK-293T with 1 μg of pcDNA6/TR (Life Technologies) that had been linearized by overnight digestion with *Pci*I which cuts once within the pUC origin of replication. Digested DNA was precipitated with 100% ethanol and washed twice with 70% ethanol before being resuspended in Tris-EDTA solution. Two days post-transfection, cells were plated in limiting dilution into 100-mm tissue culture plates and selected with 10 μg/mL blasticidin. Individual clones, expanded during the selection process, were transiently transfected with 50 ng of pcDNA5/TO-GFP (Invitrogen) using Lipofectamine 2000 and visually inspected the following day by direct fluorescence microscopy using an Olympus BX41 microscope. A pool of clones that suppressed GFP expression under these conditions was used in these studies.

#### ii. TRP-GFP Plasmid

HEK-293T cells with stable expression of TetR were transfected with 1 μg of TRP-GFP plasmid. Two day later, cells were plated in limiting dilution into 100-mm tissue culture plates and selected with 100 μg/mL hygromycin. Well-isolated clones were picked at random and expanded. To evaluate Dox de-repression, 2 x 10^5^ cells were seeded into 6-well tissue culture dishes and allowed to grow for two days in the absence or presence of 4 μM doxycycline (Sigma Aldrich) before being inspected for GFP expression by direct fluorescence microscopy or flow cytometry as described below.

#### iii. Sleeping Beauty Transposons

TetR expressing HEK-293T cells were transfected with transposon-donor plasmids (TRP-GFP; G-C-N; G-IE-N; G-IE-N (TR^TATA^) or G-IE-N (TR^Intron^)) at 500 ng each in combination with a second transposon encoding for expression of a puromycin resistance gene under the control of the human phosphogycerate kinase (PGK) promoter (50 ng), and an PGK-regulated SB11 transposase vector (500 ng). Alternatively, naïve HeLa cells were transfected with HA-IE-N transposons (500 ng) along with a second transposon encoding for bicistronic expression of TetR and a puromycin resistance gene (pKT2/CAGS-TetR-ires-Puro; 50 ng) and the SB11 transposase (PGK-SB11; 500 ng). Two days after transfection, cells were plated at limiting dilution into 100-mm tissue culture plates and selected with 0.5 μg/mL puromycin. Well-isolated clones that emerged after 10–12 days of growth were either picked at random or selected based on expression of NGFR following immunofluorescence staining and visual inspection using a fluorescent microscope. To evaluate de-repression/induction, 2 x 10^5^ cells were seeded into 6-well tissue culture dishes and allowed to grow for two days in the absence or presence of 4 μM doxycycline before being transduced overnight with adenovirus particles that conferred expression of VP16 [15] at a multiplicity of infection (m.o.i.) of three. AdVP16 titer was 1x10^9^ PFU/mL as determined by plaque forming assay [15]. Treated cells were inspected by fluorescence microscopy, flow cytometry or western immunoblot as described below.

### Fluorescence detection

Cell lines engineered for inducible expression of GFP either alone or in combination with NGFR were visualized by direct fluorescence microscopy or selected by screening clones for coexpression of NGFR by immunofluorescence staining. To detect surface levels of NGFR, cultured cells were reacted overnight with mouse anti-human NGFR p75 monoclonal antibody (ME20.4, Santa Cruz Biotechnology) and goat anti-mouse AlexaFluor 594 secondary (Life Technologies) at a 1:10,000 final dilution for each. GFP or NGFR positive cells were identified and photographed using an Olympus BX41 microscope equipped with Olympus DP70 digital camera (Olympus America) with images captured at equivalent exposure times.

### Flow cytometry

Cells were harvested with trypsin and evaluated for expression of GFP alone or when reacted with mouse anti-human NGFR p75 monoclonal antibody (ME20.4, Santa Cruz Biotechnology) and Alexa Fluor 594 conjugated goat anti-mouse H+L IgG (Life Technologies); mouse anti-human IgG was used as an isotype control. The mean of fluorescence intensity (MFI) was determined for each by flow cytometry (FACSCalibur; BD Biosciences) following collection of a minimum of 10,000 events using CellQuest v5.2.1 software (BD Biosciences). Post collection data analysis was performed with FlowJo v10.0. Values are plotted as mean + s.e.m.

### Western immunoblot

Cells were removed from plates with trypsin, washed with PBS and proteins extracted using M-PER (Thermo Scientific) supplemented with Halt Protease inhibitor cocktail (Thermo Scientific). After a 30-minute incubation on ice, samples were centrifuged (14,000 rpm/30 min/4°C), supernatants collected and protein concentrations determined by Pierce BCA Protein Assay (Thermo Scientific). Proteins (10 μg) were boiled in 2x Laemmli sample buffer (Sigma Aldrich) for 5 minutes, electrophoresed through a 10% Tris-HCl polyacrylamide-SDS gel and transferred to Immobilon-P membrane (Millipore). The membrane was blocked for 1–2 hours with 5% skim milk in Tris buffered saline with 0.1% Tween-20 (TBST). HA was detected using rabbit polyclonal antibody (1:5,000, H1N1 (A/Puerto Rico/8/1934), Sino Biological Incorporated) and TetR detected using mouse monoclonal antibody (1:1,000, Clontech Laboratories). After washing with TBST, the membrane was incubated for 1–2 hours at room temperature with horseradish peroxidase conjugated goat anti-rabbit or anti-mouse H+L IgG (all from Thermo Scientific) diluted 1:1,000 in TBST. Glyceraldehyde-3-phosphate dehydrogenase (GAPDH) was detected using a 1:75,000 dilution of monoclonal anti-GAPDH peroxidase antibody (clone GAPDH 71.1, Sigma Aldrich). Membranes were incubated with enhanced chemiluminescent (ECL) substrate (Thermo Scientific), exposed to X-ray film (CL-Xposure film, Thermo Scientific) and developed using the Konica SRX-101 developer (Konica Minolta Medical Imaging) to visualize proteins.

### Statistical analysis

Microsoft Excel software package was used to determine descriptive statistics (mean + s.e.m) and significant differences between mean values determined by Student’s *t*-test (two-tailed). *P-values* are indicated by asterisks in the figures with level of significance reported.

## Results

### Limitations of a tetracycline inducible expression system following stable gene delivery

**Fig 1:**
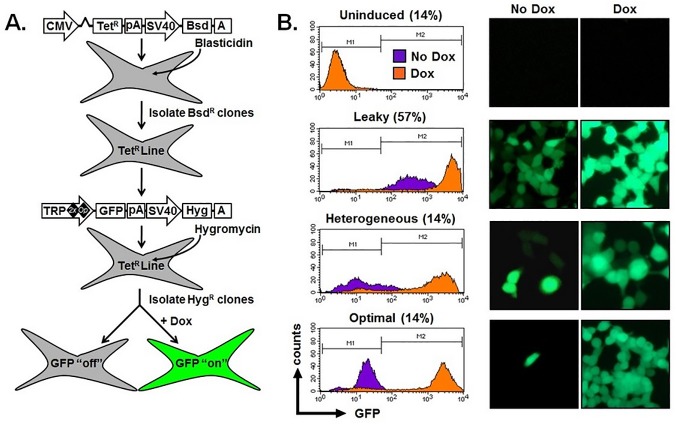
Failure to consistently generate clonal cell populations with an “off/on” phenotype using a commercially available tetracycline inducible expression system. (A) Schematic diagram of the plasmids and methodology used to create blasticidin-resistant (Bsd^R^) cell lines with stable expression of the tetracycline-repressor (Tet^R^) protein. These cells were further modified for coexpression of a hygromycin-resistance gene (Hyg^R^) and enhanced green fluorescent protein (GFP) that is repressed “off” but can become transcriptionally active “on” upon administration of doxycycline (Dox). (B) Flow cytometry histograms (left) and fluorescent microscopy images (right, original magnification 40x) demonstrating expression of GFP in cell lines cultured in the absence (purple, No Dox) and presence of 4 μM doxycycline (orange; Dox). Shown are representative examples of clones that were not inducible (Uninduced), were not adequately repressed (Leaky), were only partially induced (Heterogenous), or considered to be optimally repressed and induced (Optimal). The percentage of clones for each category are indicated (n = 21 cell lines). CMV, cytomegalovirus; Tet^R^, tet-repressor protein; TRP, tet-responsive promoter; Bsd, blasticidin resistance gene; Hyg, hygromycin resistance gene; GFP, green fluorescent protein; pA, poly adenylation signal; A, poly adenylation signal.

We first tested the effectiveness of a commercially available inducible vector (T-REx; Life Technologies) for controlled gene expression in response to de-repression by Dox. We created a cell line with stable expression of a tetracycline repressor protein (TetR) by transfecting human embryonic kidney cells (HEK-293T) and selecting for resistance to the co-expressed blasticidin resistance gene (Fig 1A). This TetR expressing line was subsequently transfected with a vector encoding for GFP under the control of a Tet-regulated version of the hCMV promoter (termed TRP 2xOP). Cells were selected for resistance to the co-expressed hygromycin gene, and twenty-one, well-isolated clones expanded and inspected for GFP expression by flow cytometry and fluorescence microscopy when grown in the absence or presence of Dox (Fig 1A).

Promoter function was evaluated using two criteria which we considered to be representative of optimal performance: (i) Repressed (No Dox), >60% of the cell population was GFP negative with a mean fluorescence intesity (MFI) <50, selected as a threshold because this level of fluorescence is below the limits of detection when cells are visualized with a fluorescence microscope; and (ii) Activated/De-repressed (Plus Dox): >80% of the cell population was GFP positive demonstrating an average 10-fold increase in MFI. Based on these criteria, generated cell lines could be placed into four categories: (i) Uninduced: no increase in MFI following addition of Dox; (ii) Leaky: initial MFI (No Dox) > 50; (iii) Heterogenous: <50% of the cell population demonstrating a 10-fold increase in expression of GFP following addition of Dox; (iv) Optimal: initial MFI (No Dox) <50 where the activated (plus Dox) MFI is >10-fold the initial level and observed in the majority (at least 80%) of the cell population. Using these criteria, the majority of clones (12 of 21) showed leaky GFP expression, such that even in the absence of Dox, GFP was expressed at levels easily detectible by flow cytometry and fluorescence microscopy (Fig 1B). The remaining nine clones were equally divided among uninduced, heterogeneous, or optimal groups (Fig 1B). MFI of GFP expression without and with Dox, fold induction, percent of cells induced by Dox treatment, and frequency for each indicated categories are provided in Table 1.

**Table 1:** Phenotypes of cell lines generated using a commercially available Tet-On vector system and random integration.

	GFP Expression for Clonal Cell Populations			
Criteria	Leaky	Uninduced	Heterogeneous	Optimal
No Dox (MFI)	**130 ± 48**	3 ± 0.1	23 ± 13	**37 ± 6**
Plus Dox (MFI)	2360 ± 295	**4 ± 0.2**	533 ± 370	**2256 ± 131**
Fold Induction	27 ± 4	1 ± 0.1	19 ± 5	**67 ± 15**
% Induced^a^	85 ± 5	0	**27 ± 19**	**89 ± 2**
# of Clones/Total Clones	12/21	3/21	3/21	**3/21**
Frequency (%)	57	14	14	**14**

^a^percent of doxycycline induced cells per clone.

MFI, mean fluorescence intensity. Values for No Dox, Plus Dox, and Fold Induction are mean ± s.e.m.

**Fig 2:**
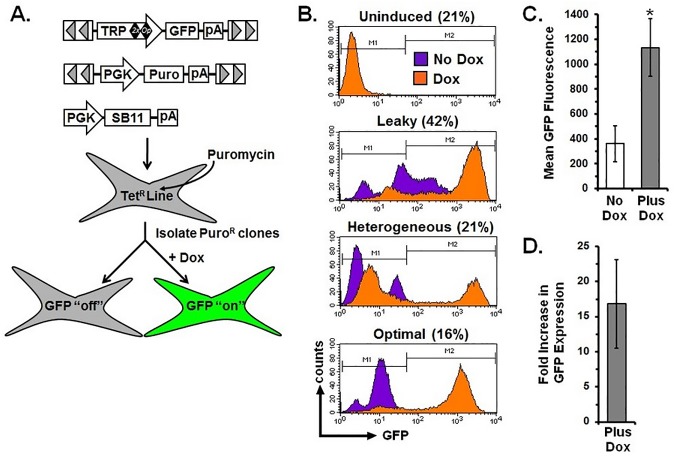
Transposon-mediated delivery of the tetracycline inducible cassette does not improve the ability to generate clonal cell populations with an “off/on” phenotype. (A) *Sleeping Beauty* (SB) transposons encoding for inducible expression of GFP (TRP-GFP) or resistance to puromycin (PGK-Puro) were cotransfected with SB transposase (PGK-SB11) into HEK-293T expressing the tetracycline-repressor protein (Tet^R^) to create cell lines that express GFP upon administration of doxycycline (Dox). (B) Flow cytometry histograms demonstrating expression of GFP in cell lines cultured in the absence (purple, No Dox) or presence (orange, Dox) of 4 μM doxycycline. Shown are representative examples of clones that were Uninduced, Leaky, Heterogenous, or Optimal with the percentage of clones for each group indicated. (C) GFP fluorescence intensity in the repressed (No Dox) and de-repressed (Plus Dox) states. *P = 0.0074 using Student’s *t*-test when compared to repressed (No Dox). (D) Fold increase in GFP fluorescence intensity when de-repressed (Plus Dox). Graphical representations of GFP fluorescence and fold increase were calculated from 19 clonal lines and reported as mean + s.e.m. TRP, Tet-responsive promoter; PGK, human phosphoglycerate kinase promoter; GFP, green fluorescent protein; SB11, *Sleeping Beauty* transposase; Puro, puromycin resistance gene; Tet^R^, Tet-repressor protein; pA, poly adenylation signal.

The aforementioned cell lines were created by plasmid transfection and subsequent selection for the co-expressed hygromycin marker. This process requires random, non-homologous recombination in the host cell genome, which is inefficient, imprecise and influenced by genomic positional effects [16]. We envisioned that transposon-mediated gene transfer could address many of these limitations and increase the number of clones that met the criteria for optimal Dox repression/de-repression (i.e., clones that display low/undetectable GFP expression basally but robust GFP expression following Dox treatment). To this end, we introduced the Tet regulated promoter-GFP-poly A cassette from the T-REx vector into a SB transposon and cotransfected TetR expressing cells with this vector, a second transposon encoding for expression of a puromycin resistance gene, and a vector encoding the SB transposase (SB11; Fig 2A) and selected for resistance to puromycin.

We isolated nineteen clones and again screened for GFP expression in the absence and presence of Dox. However, delivery of the inducible expression system using the SB transposon proved no better at achieving optimal Dox regulated GFP expression than did simple plasmid transfection (Fig 2B; Table 2). Quantification of mean GFP fluorescence for all cell lines in the repressed and de-repressed states (No Dox: 361 + 144, versus Dox: 1134 + 231, mean + s.e.m., n = 19, Fig 2C) showed an approximately 17-fold increase in GFP levels (Fig 2D). These results indicate that this Dox-responsive vector system is capable of achieving regulated gene expression; however, the frequency of obtaining tightly regulated cell lines that meet the “optimal” condition is quite low. Thus, substantial screening and selection is required to identify these few homogenous lines, as was reported for retroviral delivery of a unidirectional Tet-regulated expression cassette [17]. Consequently, we sought to develop a novel inducible system that would greatly increase the likelihood of obtaining tightly regulated gene expression in stable cell lines at high frequency.

**Table 2:** Phenotypes of cell lines generated using a commercially available Tet-On vector system and *Sleeping Beauty* transposon-mediated integration.

	GFP Expression for Clonal Cell Populations			
Criteria	Leaky	Uninduced	Heterogeneous	Optimal
No Dox (MFI)	**796 ± 283**	12 ± 9	92 ± 29	**26 ± 16**
Plus Dox (MFI)	1709 ± 388	**17 ± 15**	1184 ± 358	**1023 ± 300**
Fold Induction	4 ± 1	1 ± 0.1	25 ± 14	**62 ± 21**
% Induced^a^	9 ± 3	0	**31 ± 11**	**90 ± 5**
# of Clones/Total Clones	8/19	4/19	4/19	**3/19**
Frequency (%)	42.1	21.1	21.1	**15.8**

^a^percent of doxycycline induced cells per clone.

MFI, mean fluorescence intensity. Values for No Dox, Plus Dox, and Fold Induction are mean ± s.e.m.

### The CMV promoter is potent but lacks bidirectional activity

**Fig 3:**
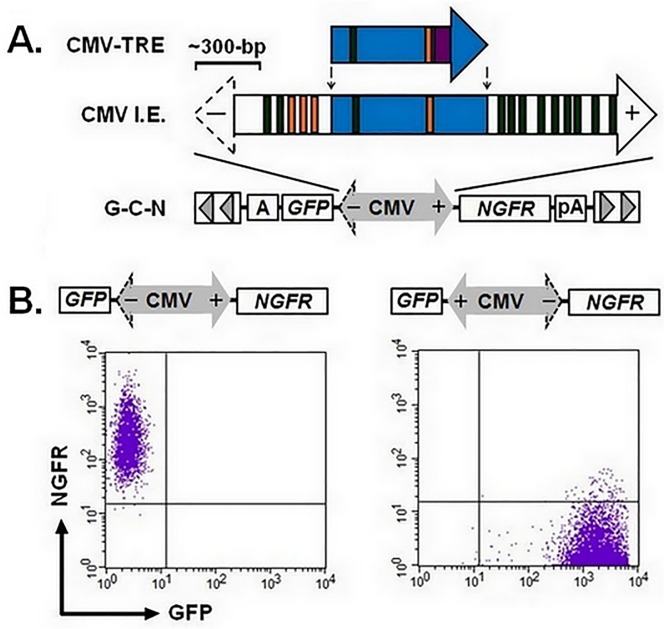
The CMV IE promoter demonstrates only unidirectional activity. (A) Schematic diagram of the inducible 728-bp CMV promoter (CMV-TRE, T-REx, Life Technologies) relative to a 2081-bp version of the CMV immediate early (IE) promoter. Closed arrow heads indicate direction of transcription, blue shading identifies consensus nucleotide sequences between the two elements where orange and green colored bars highlight canonical and non-canonical TATA boxes, respectively. The purple box for CMV-TRE identifies a 40-bp sequence encoding for two copies of a Tet-responsive element (TRE) that serve as binding sites for the Tet-repressor protein. The CMV IE sequence was inserted into a *Sleeping Beauty* transposon plasmid in between coding sequences for GFP and truncated nerve growth factor receptor (NGFR) by blunt cloning to create G-C-N. A scale bar is shown to estimate relative size of each promoter where (-) and (+) signs correspond to the reverse and forward directions. (B) Versions of G-C-N with the CMV promoter in both orientations are shown. Each version was cotransfected into human embryonic kidney (HEK-293T) cells to create puromycin-resistant cell clones (n = 5 per orientation). Representative dots plots demonstrating expression of NGFR and GFP by flow cytometry for the CMV IE promoter in forward and reverse orientations. CMV, cytomegalovirus; A, SV40 polyadenylation signal; pA, bovine growth hormone (BGH) polyadenylation signal.

We were interested in developing a system that combined a bidirectional promoter with tetracycline control elements to allow for controlled expression of a gene of interest on one side and constitutive expression of a marker gene on the opposite side to permit positive selection of stable transfectants. The CMV promoter used in the T-REx vector consists of 728-bp of core sequences from the full-length, CMV IE element (S1 Fig and Fig 3A) [13]. Bioinformatic analysis of sequences extending beyond this core region identified a number of canonical and non-canonical TATA boxes that could serve as sites of transcription initiation. Based on this analysis, we wanted to determine whether the full-length CMV IE promoter had bidirectional activity. To test this, we cloned a 2,081-bp *Pst*I-*Pst*I fragment from the CMV genome that encodes for exon 1 and the first intron of the CMV IE region I, a region that was shown to have potent promoter activity in HeLa cells [13]. Fill-in and blunt-end ligation of this fragment created SB transposons (termed G-C-N) in which the CMV promoter was positioned between an upstream GFP cassette and a downstream NGFR cassette (Fig 3A). We independently transfected naïve HEK-293T cells with G-C-N transposons in each orientation using the aforementioned three-plasmid method and selected for puromycin resistant clones. Flow cytometry analysis of the resulting cell lines demonstrated unidirectional activity for the full length CMV IE promoter with only the plus end capable of conferring GFP expression (- end: 0.5 + 0.3% of cells GFP+, MFI: 4.7 + 0.3 versus + end: 99.6 + 0.2% of cells GFP+, MFI: 2459 + 607, mean + s.e.m., n = 5 per group). This strict unidirectional activity was confirmed when cell lines were reacted with antibodies to NGFR and analyzed by flow cytometry for coexpression of this surface marker with GFP (Fig 3B). Using these two markers, we show that the CMV IE promoter exhibits transcriptional activity from only a single side evidenced by our inability to identify cells that expressed both GFP and NGFR.

### Characterization of the HSV-1 Immediate Early (IE) bidirectional promoter

**Fig 4:**
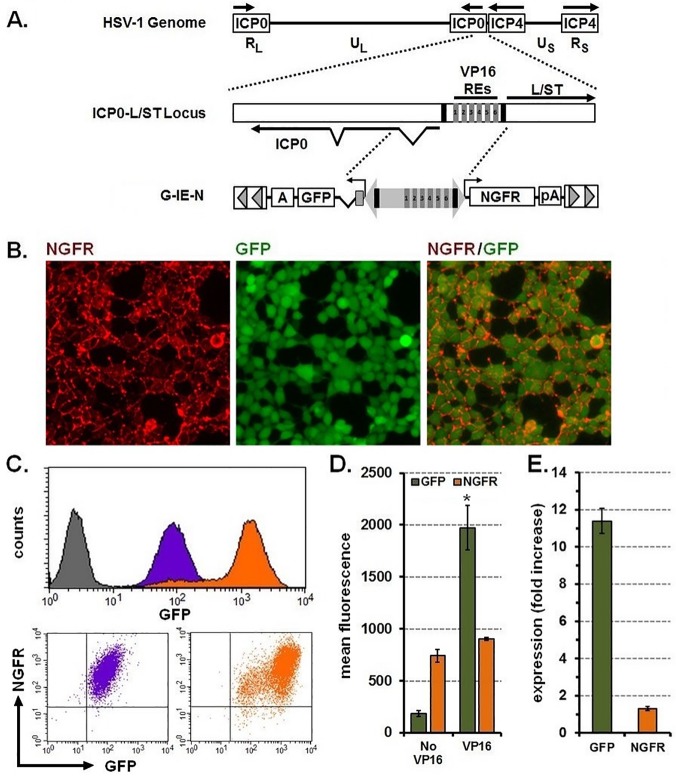
The HSV-1 IE promoter provides coordinate and constitutive expression of two genes and can be induced by VP16. (A) Top, genetic organization of the HSV-1 genome demonstrating the long-repeated (R_L_) and short-repeated (R_S_) regions that regulate expression of the immediate-early (IE) genes, infected cell proteins 0 (ICP0) and 4 (ICP4). Also indicated are unique long (UL) and unique short (US) regions that encode for most of the early and late genes. Middle, enhanced view of the IE regulatory element located between the ICP0 and long-short spanning transcript (L/ST) genes and includes six VP16 responsive elements (VP16 REs 1–6) and two TATA boxes (black bars). Bottom, 1724-bp of sequences introduced into a *Sleeping Beauty* transposon between GFP and NGFR to create G-IE-N. (B) Photomicrographs showing immunofluorescence staining for NGFR (left panel, red), direct fluorescence for GFP (middle panel, green), and merged images showing coexpression (right panel). Original magnification 40x. (C) Top, flow cytometry histograms of the clone depicted above demonstrating GFP expression before (purple) and after induction with VP16 (orange) respective of naïve controls (grey); bottom, dot plots demonstrating coordinate expression of GFP and NGFR before (purple) and after induction with VP16 (orange). (D) Mean fluorescence intensity for GFP and NGFR before (No VP16) and after (VP16) induction. *P<0.001 using Student’s *t*-test when compared to No VP16. (E) Fold increase in fluorescence intensity for GFP and NGFR after VP16 induction. Graphical representations of GFP fluorescence and fold increase were calculated from three cell lines and reported as mean + sem. VP16, transactivator of HSV immediate early gene expression; RE, responsive element; NGFR, nerve growth factor receptor; GFP, green fluorescent protein; pA, BGH polyadenylation signal; A; SV40 polyadenylation signal.

The HSV-1 genome encodes for a bidirectional promoter that directs expression of the intermediate early (IE) gene ICP0, and the L/S junction spanning transcript (L/ST) [18,19] (Fig 4A). This promoter also contains six response elements (REs) for the VP16 transactivator protein that can be used to further enhance gene expression. We replaced the full-length CMV IE promoter in the G-C-N transposon with 1724-bp of HSV-1 genomic DNA, including the six VP16 REs, such that GFP was positioned on the 5’ (ICP0) end and NGFR on the 3’ (L/ST) end to create G-IE-N (Fig 4A). To verify that this promoter had bidirectional function when removed from the HSV-1 genome, we transfected G-IE-N transposons into naïve HEK-293T cells in combination with the puromycin-encoding transposon and transposase expression vectors. Cells growing as distinct colonies were visualized for expression of GFP with all colonies (approximately 100) demonstrating some level of expression. Three clones were expanded and evaluated for expression of GFP and NGFR by immunofluorescence and flow cytometry either directly (GFP) or when reacted with antibodies to NGFR. Immunofluorescence revealed that cells exhibited NGFR on the cell surface and GFP in the cytoplasm (Fig 4B). Flow cytometry demonstrated uniform basal levels of GFP and NGFR expression (Fig 4C) where cells tended to distribute along a diagonal line in the two color dot plot, indicating coordinate expression of the two genes. Furthermore, adenoviral delivery of the VP16 transactivator enhanced GFP expression (Fig 4C and 4D: MFI: 185 + 27 No VP16 versus 1,973 + 213 VP16; mean + s.e.m., n = 3) greater than 10-fold (Fig 4E). However, a similar VP16-dependent increase was not detected for NGFR where expression levels remained generally unchanged (Fig 4C, 4D and 4E: MFI: 740 + 60 No VP16 versus 905 + 13 VP16; 1.3 fold-increase; mean + s.e.m., n = 3). Using these two markers, we confirmed that the HSV IE promoter exhibits coordinate, bidirectional activity when removed from the HSV-1 genome and stably integrated into chromosomes of mammalian cells.

### Modification of the HSV bidirectional promoter to make transcription dependent on the binding of a transactivating protein

GFP and NGFR expression analysis revealed that the HSV IE promoter demonstrated constitutive gene expression from its 3’ end and allowed for VP16-inducible gene expression from its 5’ end (Fig 4C and 4D). To provide an additional level of control from the HSV IE bidirectional promoter, we modified the VP16 inducible 5’ end to include two tandem copies of Tet operator sequences (2xOp). Because placement of the Tet operator sequences could impact basal promoter activity, we created versions of the IE promoter which contained the 2xOp sequences either near the TATA box at the 5’ end (G-IE-N(TR^TATA^)) or within the non-coding intron located immediately upstream of GFP (G-IE-N(TR^Intron^); S2A Fig). Puromycin-resistant cell lines were created by transfecting naïve HEK-293T cells with the parental G-IE-N transposon and versions that included 2xOp sequences using our three plasmid delivery method. Clones generated from each combination were screened for GFP and NGFR expression by flow cytometry. The location of the 2xOp sequences on the 5’ end of the promoter had no effect on expression of NGFR (MFI: 870 + 87 (G-IE-N), 1147 + 323 (TR^TATA^), 823 + 111 (TR^Intron^), mean + s.e.m., n = 5 for each) but did significantly reduce GFP expression when placed in the intron (GFP MFI: 233 + 41 (G-IE-N), 161 + 65 (TR^TATA^), 16 + 4 (TR^Intron^), mean + s.e.m., n = 5 for each) (S2B and S2C Fig). Therefore, the optimal placement of the 2xOp sequence is within the HSV IE bidirectional promoter at the 5’ end near the TATA box.

### The inducible HSV IE bidirectional promoter is tightly regulated and allows for controlled gene expression across a broad range of levels

We wanted to determine if transcriptional activity of G-IE-N(TR^TATA^) was dependent on the binding of the transactivating protein. To this end, HEK-293T TetR expressing cells were transfected by our three-plasmid protocol and selected for puromycin resistance. This time, generated colonies were reacted with NGFR antibodies and screened by immunofluorescence microscopy. Fourteen NGFR positive clones were evaluated for expression of GFP by flow cytometry in the absence and presence of Dox with results summarized in Table 3. Here, counter-selection for the constitutively expressed NGFR marker significantly improved our ability to identify “optimal” clones with 50% of cell lines meeting these criteria (Table 3; S3 Fig) versus only 16% (3 out of 19, see Table 2) using a commercial system lacking this co-expressed reporter. These results indicate that the inducible, HSV IE bidirectional promoter can increase the likelihood of obtaining clones with regulated gene expression.

**Table 3:** Phenotypes of cell lines generated using the inducible HSV1-IE bidirectional promoter and *Sleeping Beauty* transposon-mediated integration.

	GFP Expression for Clonal Cell Populations			
Criteria	Leaky	Uninduced	Heterogeneous	Optimal
No Dox (MFI)	**101 ± 10**	12 ± 2	38 ± 6	**14 ± 3**
Plus Dox (MFI)	473 ± 21	**28 ± 11**	177 ± 73	**208 ± 56**
Fold Induction	5 ± 0.3	3 ± 2	5 ± 1	**15 ± 2**
% Induced^a^	20 ± 0.1	32 ± 22	**40 ± 1**	**89 ± 3**
# of Clones/Total Clones	2/14	3/14	2/14	**7/14**
Frequency (%)	14.3	21.4	14.3	**50**

^a^percent of doxycycline induced cells per clone.

MFI, mean fluorescence intensity. Values for No Dox, Plus Dox, and Fold Induction are mean ± s.e.m.

To verify that variability in TetR expression was not the cause of expression differences seen among the various vectors, we performed western blots with an anti-TetR antibody. First, we assessed whether optimal clones from different vectors displayed similar levels of TetR protein. Two “optimal” clones from TRP-GFP and G-IE-N (TR^TATA^) cell lines were selected for analysis. Each clone demonstrates similar levels of TetR protein (S4A Fig). Furthermore, we assessed TetR levels in seven clonal cell lines generated using G-IE-N (TR^TATA^) that displayed either no induction (n = 1), leaky (n = 2), heterogeneous (n = 2), or optimal (n = 2) characteristics in the repressed and de-repressed states. Western blot demonstrated similar TetR protein levels for each condition (S4B Fig), indicating that TetR expression is not responsible for transgene regulation differences.

**Fig 5:**
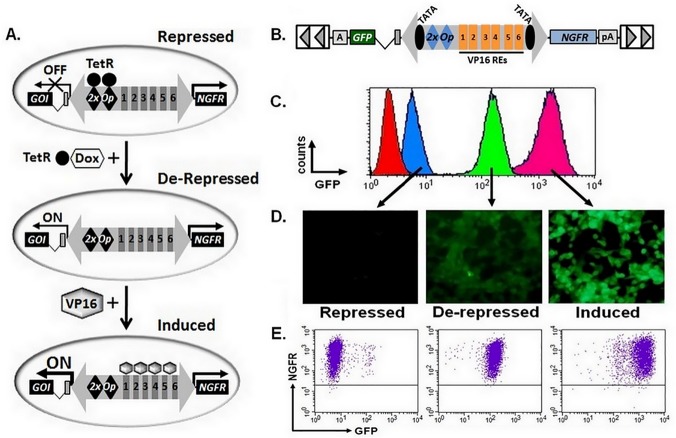
The Tet-responsive HSV-IE promoter is tightly controlled and provides a broad range of gene expression. (A) Diagram of controlled and dynamic changes in gene expression levels achieved using the modified HSV IE bidirectional promoter. Transposon gene transfer is used to simultaneously create a cell line with: i) stable expression of the tetracycline repressor protein (TetR; filled circle) and ii) constitutive expression of NGFR coupled with inducible expression of the gene of interest (GOI) controlled by the inducible IE bidirectional promoter. In the repressed state, TetR proteins bind to the target sequences (2xOp) and inhibit transcription of the GOI (OFF). TetR is inhibited upon addition of doxycycline (Dox) and transcription activated (De-Repressed, ON). Transcriptional activity of the IE promoter and expression of the GOI can be further enhanced, only for de-repressed cells, upon expression of VP16 transactivator (Induced). (B) Schematic diagram of SB transposon vectors encoding for the HSV-IE promoter with tandem copies of tetracycline-repressor target sequences (2xOp) introduced near the TATA site (G-IE-N (TR^TATA^). (C) Overlay of flow cytometry histograms demonstrating GFP expression for control cells (red), or a representative cell line cultured in the absence of doxycycline (blue, repressed), presence of doxycycline (green, de-repressed), or when doxycycline treated cells were transduced with adenovirus vector particles (m.o.i. = 3) encoding for expression of VP16 (pink, induced). (D) Fluorescent microscopy images of the same cell lines demonstrating GFP expression in the indicated states. (E) Dot plots for the same clone demonstrating coexpression of GFP and NGFR by flow cytometry in the repressed, de-repressed and induced states. VP16, trans-activator of HSV immediate early gene expression; RE, responsive element; NGFR, nerve growth factor receptor; GFP, green fluorescent protein; pA, BGH polyadenylation signal; A; SV40 polyadenylation signal.

Two cell lines created using G-IE-N (TR^TATA^) that met the “optimal” criteria were tested for de-repressive and inducible properties using a combination of Dox and VP16. Fig 5 diagrams the generation of cell lines using HSV IE bidirectional promoter system showing how expression is controlled using doxycycline and VP16 (Fig 5A). A diagram of the vector (Fig 5B) and representative example of GFP expression when clones were repressed (No Dox or No Dox, Plus VP16), de-repressed (Plus Dox) or induced (Plus Dox, Plus VP16) and evaluated for by flow cytometry (Fig 5C) or direct fluorescence microscopy (Fig 5D) or for coexpression of GFP and NGFR by flow cytometry (Fig 5E). Results for three independent experiments using both clones are provided in Table 4. Cells exhibited limited GFP expression in the absence of Dox and with or without VP16 (repressed). The addition of Dox de-repressed GFP expression to levels 9-fold over the repressed state, while the combination of both Dox and VP16 further increased GFP expression an additional 9-fold (induced; Fig 5C and 5D; Table 4); levels of NGFR were essentially unchanged for all conditions (Fig 5E, Table 4). These results demonstrate the broad range of gene expression possible from the 5’ end of the HSV IE bidirectional promoter while maintaining a constant level of NGFR expression from the 3’ end of the promoter.

**Table 4:** Gene expression levels achieved using the inducible, HSV IE bidirectional promoter.

	GFP and NGFR MFI for Clonal Cell Populations			
Treatment	GFP	Fold-Increase	NGFR	Fold-Increase
No Dox	13 ± 4	——	801 ± 95	——
No Dox, Plus VP16	33 ± 14	2.6 ± 0.42	774 ± 30	1.0 ± 0.04
Plus Dox	119 ± 15	9.1 ± 0.13	883 ± 36	1.1 ± 0.04
Plus Dox, Plus VP16	1091 ± 96	83.9 ± 0.09	792 ± 40	1.0 ± 0.05

MFI, mean fluorescence intensity. Values are mean ± s.e.m., n = 3 experiments for two independent cell lines.

**Fig 6:**
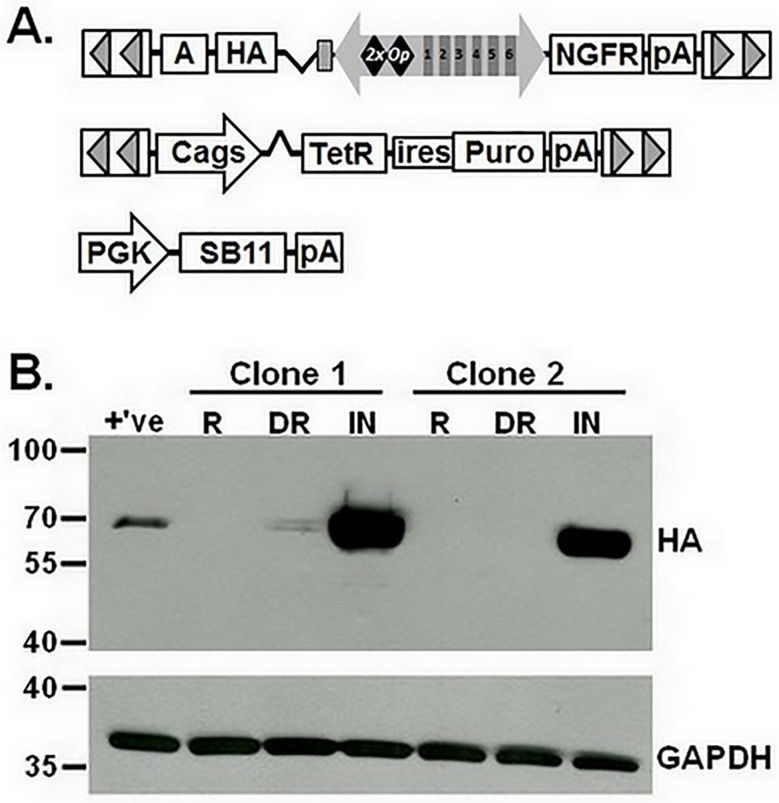
Improved utility of the inducible, bidirectional system by transposon delivery of the tetracycline repressor protein. (A) *Sleeping Beauty* (SB) transposons encoding for inducible expression of influenza A virus hemagglutinin (HA) gene (HA-IE-N) or bicistronic expression of the tetracycline-repressor (TetR) and puromycin resistance gene (Puro) were cotransfected with SB transposase (PGK-SB11) into HeLa cells to create cell lines with regulated levels of HA. (B) Western blot of total cell lysates prepared from two cell lines cultured in the absence of doxycycline (repressed, R), presence of 4 μM doxycycline (de-repressed, DR), or when doxycycline treated cells were transduced with adenovirus vector particles (m.o.i. = 3) encoding for expression of VP16 (induced, IN). Membranes were reacted with antibodies to HA or GAPDH, which served as a loading control. Molecular weights (kDal) are indicated. PGK, human phosphoglycerate kinase promoter; Cags, chimeric CMV enhancer:chicken beta-actin promoter; ires, internal ribosome entry site; pA, BGH polyadenylation signal; A; SV40 polyadenylation signal.

To verify that the inducible, bidirectional promoter could efficiently drive expression of a biologically relevant gene, we replaced GFP in G-IE-N(TR^TATA^) with sequences encoding the influenza A virus hemagglutinin (HA) gene to create HA-IE-N (Fig 6A). HA is a viral envelope protein that serves in mediating viral entry to target cells, causes red blood cell agglutination, and is used frequently as a molecular tag on exogenous protein expression. To improve the utility of our system, we created a transposon that conferred bicistronic expression of TetR and puromycin resistance from the Cags promoter [20] and cotransfected HeLa cells with this vector, HA-IE-N and the SB transposase (Fig 6A). After selecting for puromycin resistance, colonies were reacted with NGFR antibodies and screened by immunofluorescence microscopy. Two NGFR positive clones were evaluated for expression of HA by Western blot, which revealed that HA protein was undetectable under basal conditions, detectable with Dox de-repression and substantially enriched with the combination of Dox and VP16 (Fig 6B). These results demonstrate that this novel vector is capable of efficiently driving dual gene expression from a single promoter that allows for constant expression of the NGFR reporter and inducible, broad-range expression of a gene of interest. Furthermore, this vector achieves high transfection efficiency when compared to currently commercially available vectors to facilitate rapid identification of positively transfected cells.

## Discussion

We have modified a naturally occurring bidirectional promoter of the HSV-1 genome to achieve controlled and dynamic changes in gene expression using a combination of both repressor and activator elements. This promoter confers constitutive gene expression on the down-stream side, where we have introduced the NGFR gene to conveniently identify stably transfected cells using fluorescence microscopy or flow cytometry. Regulatable gene expression is possible on the upstream side of the promoter, with gene expression repressed and either “off” or at very low levels, de-repressed or “on” in the presence of a tetracycline-family drug, or induced in the presence of drug and VP16 (Fig 5A). Activity of the HSV promoter is moderate without VP16 and maximal with VP16 such that the most active “induced” state provides for a ~100-fold increase in protein levels when compared to the repressed condition. While adenovirus was used here to deliver VP16 and confer maximal activity to the HSV promoter, transfection of plasmid or *in vitro* transcribed mRNA could be used as effective alternatives [12]. Delivery of this expression system using the *Sleeping Beauty* transposon allowed for efficient development of cell lines that met these criteria particularly when the Tet repressor protein was supplied with a second transposon. The result is a novel bidirectional promoter that may be easily delivered into mammalian cells to create stable cell lines capable of tightly and uniformly controlling gene expression from levels that are essentially “off” to uniformly “on” via a combination of doxycycline-sensitive de-repression and VP16-mediated sequence-specific induction.

Coordinate gene expression is a desired trait for gene transfer applications where a gene of interest can be co-expressed with a marker or drug-selectable gene to facilitate enrichment/selection of positively engineered cells, a cytotoxic gene that allows for targeted removal of engineered cells, or shRNA sequences directed to knockdown overexpressed tumorigenic genes. A number of strategies have been employed to achieve expression of both a gene of interest and a reporter using a single vector. These strategies include dual promoters, where one promoter confers expression of the gene of interest and second promoter drives reporter gene expression [21]; gene fusion, where the gene of interest and reporter are physically linked [22]; or various read-through techniques such as internal ribosomal entry sites (IRES) (reviewed in [23]) and the Foot and Mouth Disease Virus 2A peptide or derivatives (reviewed in [24]). However, each strategy suffers from a number of limitations that restrict their usefulness [5,21,25–28]. The use of bidirectional promoters has been espoused as a better alternative for dual gene expression, as bidirectional promoters do not suffer from many of the limitations seen with the previously described systems. Most researchers have employed synthetic bidirectional promoters in attempts to achieve coordinated expression of two independent genes from a single vector. For example, Amendola and colleagues fused a minimal CMV promoter to fragments of the human PGK and ubiquitin C promoters, in opposite orientation, in a lentiviral vector and demonstrated coordinated reporter gene expression [6]. While coordinate expression of both genes was achieved, gene expression remained at a fixed amount and likely dependent on promoter choice and cell or tissue-specific context [6]. Alternatively, endogenous bidirectional promoters derived from human genomic DNA have also been used to direct dual gene expression [7], but also lack any dynamic range of expression.

Using the HSV IE bidirectional promoter to constitutively express the NGFR reporter gene, we were able to increase the likelihood of obtaining clones with regulated gene expression compared with a commercially available unidirectional, CMV-based system. Even with this improvement, we did identify clones that displayed expression characteristics that were less than optimal. Possible explanations for this include variation in levels of the TetR protein or copy number of integrated vectors encoding for the gene of interest. However, western blot analysis of TetR expression did not appear to correlate with expression characteristics in this study (S4B Fig). Copy number variation is also unlikely to explain these differences since NGFR expression was consistent among all clones generated using the G-IE-N(TR^TATA^) vector (Table 3, S2B and S2C Fig). We attribute the uniform expression of NGFR to the use of the SB transposon for gene delivery. For example, Turchiano and colleagues demonstrated a strong correlation between copy number and transgene expression in clonal cell lines created using *Sleeping Beauty* [29], suggesting that clones created with the HSV IE vector would have similar numbers of integrants per cell.

Bidirectional promoters are common throughout nature and are estimated to comprise approximately 10% of human protein coding genes (reviewed in [30]). These promoters frequently confer coordinate expression of the regulated genes, which often participate in the same biological pathway, such as DNA repair [31]. A number of authors have proposed that bidirectional activity may be a common feature of many promoters (reviewed in [32]). Bioinformatic approaches have identified differences in the genomic structures of unidirectional and bidirectional promoters [30,32] which may allow prediction of whether a given promoter possesses bidirectional function. Notably, bidirectional promoters frequently exhibit higher GC (>60%) content than unidirectional promoters. While the HSV IE and CMV IE promoters are both members of the herpes virus family (*Herpesviridae*), only the HSV IE promoter is capable of bidirectional activity. Interestingly, the HSV IE promoter has a GC content of 68% while the full length CMV promoter has a GC content of 47.7% and the truncated (commercially available) CMV promoter has a GC content of 48.4%. Furthermore, a CpG island search [33,34] revealed extensive CpG island structure for the HSV IE promoter but none for the CMV promoter. This suggests that a similar genomic organization of bidirectional promoters exists in humans and viruses.

Inducible control of exogenous gene expression is often desirable to allow for fine-tuning of the quantitative and/or temporal levels of a gene of interest. Components of the tetracycline repressor are most often employed in inducible vector systems due to its simplicity, ease of use, and rapid gene induction. However, Tet-regulated systems can be “leaky”, that is, they may allow some level of gene expression even in the absence of the inducer (see Figs 1B and 2C) [1,16]. Furthermore, Tet-regulated vectors can allow for graded de-repression that typically occurs over a narrow molar range [35,36] (data not shown). Our system has several key features versus currently available inducible vectors. First, we incorporated two Tet-response elements into the endogenous HSV bidirectional promoter to permit gene expression in a tightly regulated manner. Gene expression following addition of Dox is homogenous averaging nearly 10-fold above background and likely within the range of housekeeping genes. Second, use of the HSV bidirectional promoter, with its naturally occurring VP16 response elements, provides for a second degree (~10-fold above dox de-repression) of gene expression to further regulate final protein levels. Third, the bidirectional nature of the HSV promoter allows for expression of a second gene to be unaffected when cells are treated with Dox or with Dox and VP16. This is advantageous when the second gene is a reporter gene (in our case NGFR) where consistent expression is necessary for accurate assessment of gene transfer and to easily select for cells with the repressed or “off” phenotype. Our data demonstrate that NGFR and the gene of interest are co-expressed in the same cell, confirming the validity of the reporter gene as an indicator of gene of interest expression. Finally, this system would be adaptable to a technology used to create viral vectors to expand the range of cells available for manipulation. These collective characteristics address major limitations of current methods and provide an excellent strategy to investigate the effects of gene dosing in any mammalian model.

## Supporting Information

S1 Fig Cytomegalovirus immediate early gene promoter. Sequence of a 2,081-bp *Pst*I-*Pst*I fragment of the cytomegalovirus (CMV) genome represented from 5’- to 3’-direction. Bold, italic sequences are consensus TATA boxes; bold sequences are non-canonical TATA boxes; boxed sequences highlight the consensus CMV promoter sequence commercially available from Invitrogen where the arrowhead indicates direction of transcription.
(TIF)

S2 Fig Positional effect of tetracycline operator sequences on basal transcriptional activity of the HSV1-IE promoter. (A) Schematic diagram of the SB transposon vector encoding for the wild type IE promoter (G-IE-N, top) or versions where two tandem copies of tetracycline-repressor target sequences (2xOp) were introduced within close proximity to the transcriptional start site and located near the TATA site (G-IE-N(TR^TATA^); middle) or in the first intron (G-IE-N(TR^Intron^); bottom). Transcriptional activity of the promoter was monitored by flow cytometry analysis of NGFR and GFP expression in clonal populations of naïve HEK-239T cells. (B) Dot plots of a representative clone generated for each of the indicated constructs showing expression levels of NGFR and GFP. (C) Graphical representations of mean fluorescence intensity for GFP and NGFR calculated for five clones per vector and reported as mean + sem. *P = 0.0006 using Student’s *t*-test when compared to G-IE-N.
(TIF)

S3 Fig Tet-responsive activity of the HSV-IE promoter. Overlay of flow cytometry histograms for the seven “optimal” clones generated using G-IE-N(TR^TATA^) demonstrating GFP expression in the absence (purple, repressed, M1 gate) and presence of 4 μM doxycycline (orange, de-repressed, M2 gate). The quantified results are reported in Table 3.
(TIF)

S4 Fig TetR protein levels in engineered cells. (A) Whole cell protein lysates from either G-IE-N(TR^TATA^) or TRP-GFP (2 optimal clones each) were reacted with anti-TetR antibody or GAPDH (loading control). Control lanes show transiently transfected TetR (+’ve) and mock transfected HEK-293T cells (-‘ve). M indicates lane loaded with a protein ladder. A vertical line was inserted to represent repositioned lanes on the gel image. (B) Whole cell protein lysates from G-IE-N(TR^TATA^) clones representing different expression characteristics were reacted with anti-TetR antibody or GAPDH (loading control). O is optimal; H is heterogeneous; L is leaky; and NI is not inducible. Mock transfected HEK-293T cells (-‘ve) served as a TetR negative control.
(TIF)
