# Supplementary figures and images for: A System for Creating Stable Cell Lines that Express a Gene of Interest from a Bidirectional and Regulatable Herpes Simplex Virus Type 1 Promoter
Source: PLoS One. 2015 Mar 30;10(3):e0122253. doi: 10.1371/journal.pone.0122253 (PMC4378986; doi:10.1371/journal.pone.0122253)

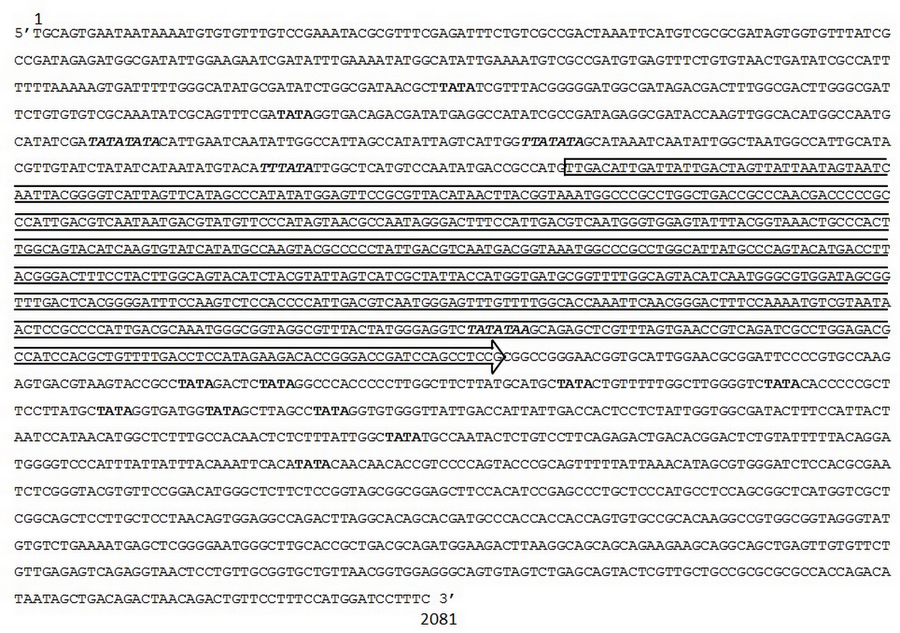

Supplement: S1 Fig — Sequence of a 2,081-bp PstI-PstI fragment of the cytomegalovirus (CMV) genome represented from 5’- to 3’-direction. Bold, italic sequences are consensus TATA boxes; bold sequences are non-canonical TATA boxes; boxed sequences highlight the consensus CMV promoter sequence commercially available from Invitrogen where the arrowhead indicates direction of transcription. (TIF) [file pone.0122253.s001.tif]

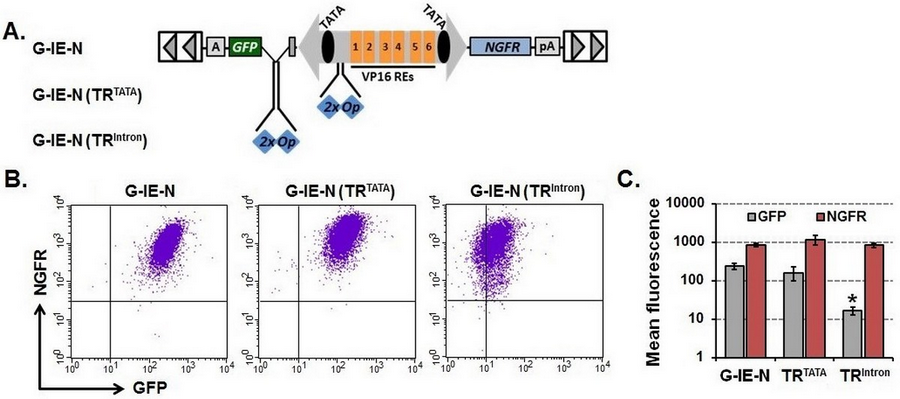

Supplement: S2 Fig — (A) Schematic diagram of the SB transposon vector encoding for the wild type IE promoter (G-IE-N, top) or versions where two tandem copies of tetracycline-repressor target sequences (2xOp) were introduced within close proximity to the transcriptional start site and located near the TATA site (G-IE-N(TRTATA); middle) or in the first intron (G-IE-N(TRIntron); bottom). Transcriptional activity of the promoter was monitored by flow cytometry analysis of NGFR and GFP expression in clonal populations of naïve HEK-239T cells. (B) Dot plots of a representative clone generated for each of the indicated constructs showing expression levels of NGFR and GFP. (C) Graphical representations of mean fluorescence intensity for GFP and NGFR calculated for five clones per vector and reported as mean + sem. *P = 0.0006 using Student’s t-test when compared to G-IE-N. (TIF) [file pone.0122253.s002.tif]

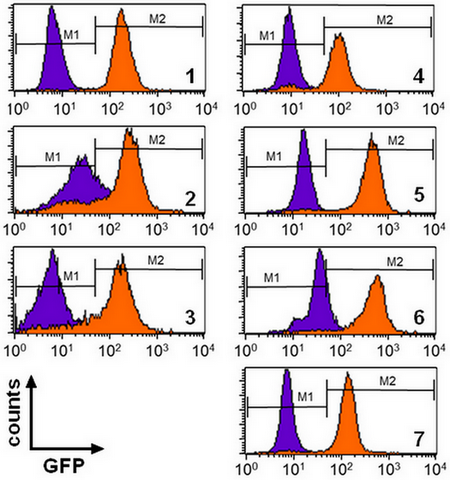

Supplement: S3 Fig — Overlay of flow cytometry histograms for the seven “optimal” clones generated using G-IE-N(TRTATA) demonstrating GFP expression in the absence (purple, repressed, M1 gate) and presence of 4 μM doxycycline (orange, de-repressed, M2 gate). The quantified results are reported in Table 3. (TIF) [file pone.0122253.s003.tif]

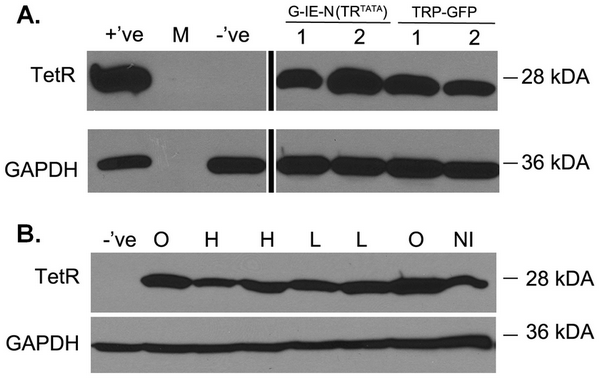

Supplement: S4 Fig — (A) Whole cell protein lysates from either G-IE-N(TRTATA) or TRP-GFP (2 optimal clones each) were reacted with anti-TetR antibody or GAPDH (loading control). Control lanes show transiently transfected TetR (+’ve) and mock transfected HEK-293T cells (-‘ve). M indicates lane loaded with a protein ladder. A vertical line was inserted to represent repositioned lanes on the gel image. (B) Whole cell protein lysates from G-IE-N(TRTATA) clones representing different expression characteristics were reacted with anti-TetR antibody or GAPDH (loading control). O is optimal; H is heterogeneous; L is leaky; and NI is not inducible. Mock transfected HEK-293T cells (-‘ve) served as a TetR negative control. (TIF) [file pone.0122253.s004.tif]
